# Explaining Human Comparisons Using Alignment-Importance Heatmaps

**DOI:** 10.1007/s42113-025-00235-x

**Published:** 2025-03-11

**Authors:** Nhut Truong, Dario Pesenti, Uri Hasson

**Affiliations:** Center for Mind/Brain Sciences (CIMeC), University of Trento, Rovereto, Trento 38068 Italy

## Abstract

We present a computational explainability approach for human comparison tasks, using Alignment Importance Score (AIS) heatmaps derived from deep-vision models. The AIS reflects a feature map’s unique contribution to the alignment between deep neural network’s (DNN) representational geometry and that of humans. We first validate the AIS by showing that the prediction of out-of-sample human similarity judgments is improved when constructing representations using only higher-scoring AIS feature maps identified from a training set. We then compute image-specific heatmaps that visually indicate the areas that correspond to feature maps with higher AIS scores. These maps provide an intuitive explanation of which image areas are more important when it is compared to other images in a cohort. We observe a correspondence between these heatmaps and saliency maps produced by a gaze-prediction model. However, in some cases, meaningful differences emerge, as the dimensions relevant for comparison are not necessarily the most visually salient. To conclude, Alignment Importance improves the prediction of human similarity judgments from DNN embeddings and provides interpretable insights into the relevant information in image space.

## Introduction

### The Question: Explaining Human Comparisons

Work in recent years has shown that DNNs learn feature spaces whose geometry has some similarity to that of humans. This is convincingly shown by the fact that human similarity judgments (HSJs) for pairs of words or images are often quite well predicted by the distances between image pairs or word pairs in vision-DNNs or language models (for reviews, see Battleday et al., 2021; Roads & Love, 2024; Sucholutsky et al., 2023). These models therefore naturally extract features relevant for modeling HSJs when trained on standard tasks such as image classification or word prediction. While the object embeddings of such pretrained machine learning models approximate HSJs quite well, it has been further shown that these predictions can be considerably improved using down-stream operations.

One such operation is to learn a reweighting of the products of feature values, which improves prediction of HSJs for both images (e.g., Peterson et al., 2018; Kaniuth & Hebart, 2022) and words (e.g., Richie & Bhatia, 2021). Another approach is to use supervised pruning to assess features’ importance in the context of estimating a set of similarity judgments (Tarigopula et al., 2023; Flechas Manrique et al., 2023). Pruning does not alter the activation weights of the retained features, but instead removes a subset of features from the embedding matrix. Pruning has also been used to identify sub-spaces in language models that optimize particular classification tasks (e.g., Cao et al., 2021).

While prior work has shown that pruning of nodes in a DNN’s penultimate layer can improve the prediction of similarity judgments, here we are interested in its potential to explain what parts of an image matter for the judgment itself. Understanding which information is used as a basis for comparison is a fundamental question in cognitive science. Since the work of Tversky (1977), many studies have shown that comparisons between objects are a function of those elements that are shared or distinct between them. However, for naturalistic stimuli, it is difficult to know which properties are important when an image is compared to a target set of images. Here we suggest that this question is tractable via a computational solution in which latent dimensions that are related to the comparison process are identified and projected onto the image space as a heatmap.

### Logic of the Current Study

We present the logic here, with a complete formal presentation provided in the “Preliminaries” section. Our approach relies on evaluating how pruning changes the alignment between human and computer-model representational spaces. Both spaces are operationalized using pairwise distances between images. One set of distances is derived from human behavior ($HB_{dist}$), and the other is computed from a computer model ($Model_{dist}$). We define the baseline isomorphism between the two spaces as the correlation between these two vectors.

In the next step, a perturbation is introduced to the feature representations of an image. Specifically, a feature map in the last convolutional layer is masked. Therefore, the information from that feature map is not encoded in the model, and not propagated onwards to the fully connected layer from which we obtain image embeddings. Subsequently, $Model_{dist}$ is recomputed, as is the isomorphism between the representations. Note that only the target image is affected, and not the other images. Furthermore, $HB_{dist}$ remains unchanged. There are two possible outcomes: *i*) if the encoded information from the feature map is cognitively irrelevant or even confounding, its removal could alter $Model_{dist}$ in a way that improves the isomorphism with human similarity judgments. Conversely, *ii*) if the encoded information from the feature map is cognitively relevant (e.g., masking a feature map representing an animal’s face in the context of similarity judgments between animals), its removal will alter $Model_{dist}$ in a way that decreases the isomorphism with human judgments. This occurs because the way that images stand in relation to each other in the DNN representation is now lacking information that underlies human judgments. By iteratively masking all feature maps in the last convolutional layer, each feature map is linked with a perturbation score indicating its importance.

A similar logic was presented in previous studies, but in those, masking was applied to the image space rather than the latent feature space. For instance, Tarigopula et al. (2023) used this approach with human neuroimaging data to explain which parts of an image contain information relevant to the representational space of various brain regions. In other work, Palazzo et al. (2020) masked image patches to evaluate how masking impacted the compatibility between vision-DNN embeddings and EEG data.

### Current Aims and Contribution

The current study’s aims advance over prior studies in three respects: it directly studies human comparison processes, it introduces an advantageous masking procedure, and it evaluates the results against typical saliency maps. The aforementioned studies operationalized representational spaces from multivariate fMRI and EEG recordings but have not studied human comparison processes. Furthermore, the technique they use, namely, mask-sweep over an image, presents several major limitations: (1) the mask size is arbitrary, requiring the use of multiple sizes; (2) an arbitrary decision is required regarding how to combine information from different mask sizes; (3) the process is computationally costly, as masks are ideally applied with each pixel being in the mask center; and (4) a theoretical weakness is that the mask is not informed by prior information contained in the model.

Departing from these prior studies, here we directly model human comparison judgments and use a different, more efficient approach to masking images, which uses information already present in the DNNs own feature space. Specifically, we focus on the feature maps in a deep convolutional layer and use them to define the masks. Our approach is inspired by Score-CAM (Score-weighted Class Activation Maps; Wang et al., 2020) which is an explanatory method that generates heatmaps indicating which sections of a target image are relevant for its classification. Score-CAM takes the information in each feature map, upscales it to the original input resolution, uses it as an information selector for the original input image, and computes the activation for the correct class (pre-softmax confidence) when using that feature map alone. After repeating this process for all feature maps, the confidence scores are used as weights to generate a heatmap highlighting image areas important for classification. Using a similar logic, we show that information at the feature map level is also highly useful for identifying which feature maps are important for the alignment between the DNN and human representational spaces and that these can be visualized in a similar manner.

Beyond our main explainability objective, we have two other important aims. First, we evaluate whether it is possible to identify feature maps that are particularly important for predicting human representational spaces; using only these feature maps should improve out-of-sample prediction accuracy for human similarity judgments as compared to using all feature maps. Second, we evaluate the relationship between heatmaps produced using this method, and traditional saliency maps trained to predict human gaze patterns. While the latter operationalize saliency using information latent in the image itself, the heatmaps we produce highlight information pertinent to image comparisons within a given set. In this context we also evaluate the relation between our method and the information provided by Score-CAM, which is also based on assigning importance scores to each feature map.

## Methods

### Preliminaries

•  Architecture and datasets: In the main analysis, We use VGG-16, a deep neural network (Simonyan & Zisserman, 2014), pre-trained on ImageNet (Deng et al., 2009) and another trained on Ecoset^1^ (Mehrer et al., 2021). VGG-16 was used because Ecoset was trained on that model. It is also a common architecture used for predicting human similarity judgements (Peterson et al., 2018; Kaniuth & Hebart, 2022) and has been shown to be a good candidate for behavior or brain alignment (Schrimpf et al., 2018). As images we used stimuli provided by Peterson et al. (2018), which consisted of 720 images divided into six categories, each containing 120 images. The categories were Animals, Fruits, Furniture, Various, Vegetables, and Automobiles (the latter effectively including any means of transportation including horses, sleds, and cranes; Transportation henceforth). Images had a native resolution of $500\times 500$ which was downscaled to $224\times 224$ to match the model’s training data. We refer to the groups of 120 images as categories or datasets interchangeably depending on whether we refer to their semantic property or the objects (images) they contain.

•  Human Similarity Judgments: Let $\displaystyle {\boldsymbol{H}}$ be a matrix representing the similarity judgments provided by human assessors for *n* objects. Each entry $\displaystyle {H}_{i,j}$ in the matrix corresponds to the similarity judgment between objects *i* and *j*. We use the upper triangle of matrix $\displaystyle {\boldsymbol{H}}$, denoted as $\displaystyle {\boldsymbol{H}}_u$.

•  Object distances in feature space: Let $\displaystyle {\boldsymbol{C}}$ be a matrix representing the embeddings of *n* images onto *d* features of the penultimate layer of the pre-trained computer vision model, denoted as $\displaystyle {\boldsymbol{C}}\in \mathbb {R}^{n \times d}$. Specifically, we use VGG-16 with $d=4096$, and the number of images in each of the categories provided by Peterson et al. is $n=120$. Matrix $\displaystyle {\boldsymbol{C}}$ is obtained by considering all parameters of the pre-trained model, and specifically all 512 feature maps of the deepest convolutional layer. $\displaystyle {\boldsymbol{Z}}_u$ is the upper triangle of image-pair similarity matrix $\displaystyle {\boldsymbol{Z}}$, computed from the cosine similarity for each row pair in $\displaystyle {\boldsymbol{C}}$.

•  Submatrices of matrix $\displaystyle {\boldsymbol{C}}$: We produce two types of submatrices of $\displaystyle {\boldsymbol{C}}$ (all with dimension $n \times d$). The first submatrix (“remove 1”), denoted as $\displaystyle {\boldsymbol{C}}^{(\lnot k)}$, is constructed by excluding feature map *k* where $k \in \{1, 2, \ldots , 512\}$. The second type of submatrix is produced when using only a subset *S* of feature maps in the model. Let $S \subseteq \{1, 2, \ldots , 512\}$ be a set of selected feature map indices, and let $\displaystyle {\boldsymbol{C}}^{(S)}$ be the matrix representing the embedding of $n$ images onto *d* nodes in the penultimate layer, but when using the subset of feature maps corresponding to $S$. Note that in all cases, the (one or more) feature map activations are propagated to the penultimate layer using the pre-trained weights.

•  From the submatrices of $\displaystyle {\boldsymbol{C}}$ we derive matching similarity matrices. The first, $\displaystyle {\boldsymbol{Z}}^{(\lnot k)}$, is obtained by computing the cosine similarity for each pair of rows in $\displaystyle {\boldsymbol{C}}^{(\lnot k)}$. The second, $\displaystyle {\boldsymbol{Z}}^{(S)}$ is formed using the selected feature indices in $\displaystyle {\boldsymbol{C}}^{(S)}$.

•  As indicated, $\displaystyle {\boldsymbol{Z}}_u$ and $\displaystyle {\boldsymbol{H}}_u$ denote the vectorized upper triangles of matrices $\displaystyle {\boldsymbol{Z}}$ and $\displaystyle {\boldsymbol{H}}$ respectively. The Spearman correlation coefficient between the two is denoted as $\rho ({\displaystyle {\boldsymbol{Z}}_u, \displaystyle {\boldsymbol{H}}_u})$. We refer to this value as a baseline second-order-isomorphism (2OI) between the two domains. Analogously, in some cases we compute $\rho ({\displaystyle {\boldsymbol{Z}}^{(\lnot k)}_{u}, \displaystyle {\boldsymbol{H}}_u})$ and $\rho ({\displaystyle {\boldsymbol{Z}}^{(S)}_{u}, \displaystyle {\boldsymbol{H}}_u})$.

### Aim 1: Identifying a Subset of Feature Maps that Optimizes Prediction of Human Similarity Judgments

We define the Alignment Importance Score (AIS) of each feature map in terms of its predictive capacity for the human representation $\displaystyle {\boldsymbol{H}}_u$. Intuitively, we aim to determine how the removal of each feature map $k \in \{1, 2, \ldots , 512\}$ affects the baseline isomorphism, $\rho ({\displaystyle {\boldsymbol{Z}}_u, \displaystyle {\boldsymbol{H}}_u})$. The removal of each feature map produces a modified 2OI score, $\rho ({\displaystyle {\boldsymbol{Z}}^{(\lnot k)}_{u}, \displaystyle {\boldsymbol{H}}_u})$. Finally, The AIS of feature map *k* is defined in Eq. 1, with positive values indicating a relatively important feature map, and negative values a less important one. After computing AIS for all feature maps, we rank-order them based on their AIS.

1 $$\begin{aligned} \text {AIS}_{k} = \rho ({\displaystyle {\boldsymbol{Z}}_u, \displaystyle {\boldsymbol{H}}_u}) - \rho ({\displaystyle {\boldsymbol{Z}}^{(\lnot k)}_{u}, \displaystyle {\boldsymbol{H}}_u}) \end{aligned}$$

We then identify an optimal subset of feature maps for predicting $\displaystyle {\boldsymbol{H}}_{u}$. In each iteration, one feature map is added to the subset *S* in descending order of AIS rank, and we recompute the 2OI, $\rho ({\displaystyle {\boldsymbol{Z}}^{(S)}_{u}, \displaystyle {\boldsymbol{H}}_u})$ using that subset of feature maps alone. After these 512 iterations, subset $S^{*}$ ultimately selected is the one that maximizes 2OI.

To validate AIS, we use an 80:20 cross-validation framework where 80% of the entries in $\displaystyle {\boldsymbol{H}}_u$ are assigned to a training set, and the remaining 20% constitute the test set. The optimal subset of feature map indices, $S^{*}$, is determined from the training set using sequential features selection as described above. For statistical significance testing, we repeat the entire cross-validation process eight times with different dataset shuffling. This produces 40 Full vs. Retained value pairs for each relevant comparison. To evaluate generalization, we use only this $S^{*}$ set of feature maps to predict HSJs on the test set. Prediction performance is compared against a baseline where all 512 features are used for predicting HSJs in the test set. Statistical significance testing, per dataset, is based on the 40 value-pairs produced via cross-validation, which are analyzed using paired two-tailed T-tests (12 tests in all, non-corrected for multiple comparisons). The success of Aim 1 is determined if $\rho (\displaystyle {\boldsymbol{Z}}^{(S^*)}_{u}, \displaystyle {\boldsymbol{H}}_u)$ surpasses $\rho (\displaystyle {\boldsymbol{Z}}_{u}, \displaystyle {\boldsymbol{H}}_u)$, indicating superior prediction compared to the baseline using a subset of feature maps.

### Aim 2: Explaining Human Similarity Judgments

#### Computing AIS Scores for a Single Image

Our goal is to identify which image patches, in image space, are relevant to comparisons between a target image *t* and other images in the set. This is visualized by creating a heatmap for *t* identifying those image sections, as follows. We begin by defining a baseline 2OI for *t* as the Spearman correlation between the $n-1$ similarity judgments associated with *t*, as quantified from the model, and the corresponding set of human similarity judgments. As in Aim 1, we define the AIS of feature map $k$ by computing a value that reflects the departure from baseline, as indicated in Eq. 1. We iterate over all 512 feature maps, producing 512 AIS values that indicate the relative importance of each feature map for the alignment between DNN-derived distances and human similarity judgments for target image *t*. These values are used to produce the heatmaps as detailed in the “Producing Heatmaps from AIS Scores” section.

#### Producing Heatmaps from AIS Scores

Image-level heatmaps are then computed as follows. We first convert negative AIS values to zero because they indicate features that encode information less relevant to modeling the human data (see Eq. 1). The remaining scores are sum normalized. Subsequently, feature maps for an image are weighted-averaged according to their corresponding AIS to create a heatmap. In the heatmaps, warmer colors indicate image areas associated with the more important features. This algorithm for producing a heatmap for a single image is presented more formally in the Appendix.

To quantify the similarity between the heatmaps generated by Ecoset and Imagenet, we defined a Match score for each image, as the Pearson correlation between the heatmap generated by the Ecoset model and the one generated by the Imagenet model. Anticipating the results, in certain instances, the Match score was low. We, therefore, examined if this occurred for images that did not correspond to classes on which the models were trained. For each image, we computed the entropy of the post-softmax probability distributions, independently for the Ecoset and ImageNet-trained models. The higher of these two entropy values was retained and designated as maxEntropy. Subsequently, considering all images in a dataset, we computed the correlation between the Match score and maxEntropy.

We note that no learning or out-of-sample testing is involved in producing these heatmaps. After establishing that the AIS has construct validity (i.e., it effectively predicts similarity judgments; Aim 1), we compute AIS values directly for each individual image ***t*** considering the similarity of image ***t*** to all other images in the category.

#### AIS Distributions for ImageNet and Ecoset Models

Computing the 512 AIS values for each of the images in a dataset produces an $n \times k$ matrix (120 [AIS] x 512 [feature map]) for each dataset containing 120 images. We then compare these distributions between the ImageNet and Ecoset-trained models to understand if and how the training regime impacts the distribution of AIS. Histograms are computed for the mean AIS value by feature and the mean absolute deviation, computed by feature (column) and by image (row).

### Aim 3: Cross-Referencing Heatmaps Against Score-CAM and Saliency Maps

We compared the heatmaps produced by our method to those produced by two other explanatory methods, that emphasize, respectively, category-membership (Score-CAM; Wang et al., 2020), and salience (TranSalNet; Lou et al., 2022).

#### Relating to Score-CAM

**Score-CAM** (Wang et al., 2020) creates explainability heatmaps by assigning each feature map in a pre-trained model an importance score that indicates that feature map’s contribution to correct classification. It can be applied to feature maps from a VGG-16 model pretrained on ImageNet, which is the same architecture we used in Aims 1 and 2. Consequently, Score-CAM scores (Classification Importance Score; CIS) can be directly related to the AIS scores computed for the same feature maps. Thus, to determine whether our method and Score-CAM are loading on the same semantics, for each image we computed the correlation between that image’s 512 CIS and AIS values. A high correlation would suggest that for the image, image areas important for classification are also those important for explaining similarity, whereas a low correlation would indicate they load on different components. Because both methods are based on weighting the same feature maps, this comparative analysis does not require generating or evaluating heatmaps in image space, as it is sufficient to study feature map information.

Note that Score-CAM outputs are meaningful for correctly classified images. Because the images used in our study, obtained from Peterson et al., were not part of the ImageNet training set, we first needed to identify a subset that was classified correctly. We, therefore, decided to focus on the 120 Animal images, as animal categories are well represented in ImageNet, and selected only those animal images that were correctly classified. To this end, we ran inference on the 120 images using VGG-16 and obtained their top-1 labels. From these, we retained only those images ($n=57$) for which all three authors agreed the top-1 label was accurate.

For statistical analysis, we computed the correlation between AIS and CIS for each image, and reported on the distribution of the correlation scores. We produce comparative heatmaps for pairs of images for which the correlations were minimal, maximal, or belonged to the 25th, 50th, or 75th percentile points.

#### Relating to Saliency: Precision-Recall Curves

The second method, TranSalNet Lou et al. (2022), is based on a state-of-the-art DNN that identifies salient image sections and accurately predicts human gaze patterns (see Fig. 10 in Appendix). Because TranSalNet uses a custom architecture, we could not relate feature map importance as we did with Score-CAM and we therefore compared TranSalNet-produced heatmaps to those produced by our method. We cross-reference TranSalNet against our method (AIS) using two approaches: precision-recall curves and subset analyses. First, we evaluate how well a pixel’s salience predicts its inclusion in an AIS heatmap. When the salience and AIS maps are thresholded at a specified level to form binarized maps, the relationship between them can be understood in terms of precision and recall. The binarized AIS map is treated as the target variable, and the binarized saliency map is the predicting variable. In this case, we have:

$$ \text {Precision} = \frac{|TranSalNet \cap AIS|}{|TranSalNet|} $$

$$ \text {Recall} = \frac{|TranSalNet \cap AIS|}{|AIS|}. $$

We describe this relationship using a precision-recall curve. The curve is generated by thresholding the AIS map at a fixed level and then plotting precision versus recall as the saliency map is thresholded across a range of levels.

The following steps were performed for each image: first, we created a heatmap as described in Aim 2 and generated a corresponding saliency map using TranSalNet. We kept the same aspect ratio of the images input to both VGG-16 and TranSalNet for compatibility in later comparisons. We conducted four separate analyses, where we created a binary mask for the AIS map at each of the following percentiles: $P = \{60, 70, 80, 90\}$. In each analysis, we thresholded the saliency maps at all percentiles between 1 and 99, with a step size of 2. Percentiles were calculated separately for each image.

#### Relating to Saliency: Conditional Probability Analysis

In this analysis, we aim to identify whether an image section (specifically, a pixel) identified as salient (*Sal*) is more likely to also be identified as comparison-relevant (*CR*; that is, warm-colored in our analysis). To do this, we threshold both maps to select the top 5% of Salient and *CR* pixels, producing *Sal*, $\lnot Sal$, *CR* and $\lnot CR$ partitions of the image pixels. We then compute the relative risk (RR) ratio as in Eq. 2.

2 $$\begin{aligned} \text {RR} = P(CR | Sal) \div P(CR | \lnot Sal) \end{aligned}$$

The relative risk as computed here measures the likelihood of *Sal* pixels being *CR* pixels compared to $\lnot Sal$ pixels. An *RR* value greater than 1 indicates that salient pixels are more likely to be CR than non-salient ones, while an RR less than 1 indicates the opposite. A main difference between this analysis and the precision-recall one is that it also quantifies joint distributions within the non-salient pixel set. We repeat this analysis when thresholding both maps at 10% and 15% top *Sal* and *CR* pixels.

We note that there is no requirement that the two methods identify the same image features. The saliency map is driven by image features (including higher-level semantics captured by the DNNs), whereas the heatmap we produce from AIS values is a function of how a certain object stands in relation to other objects in the set. As we will see, this produces cases of very high overlap, but also important distinctions.

### Aim 4: Generalization to other Architectures and Training Objectives

In Aims 1, 2, and 3, the image embeddings used were obtained from VGG-16. VGG-16, and a later variant VGG-19, are somewhat unique in that after the deepest convolutional layer, they also include two very large fully connected layers. These layers perform non-linear, abstract interactions over the information in the deepest feature map layer, and are essential for linking this information to the classification task.

Many other computer vision architectures do not include such layers and instead use the deepest feature maps, relatively directly, for classification. This is done by implementing global average pooling, which reduces each of these feature maps into a single value, followed by learning a linear combination of these values for classification. Thus, in these architectures, the final layer before classification receives an input corresponding to the number of feature maps (after global pooling) and produces an output corresponding to the number of classes to be learned.

**Fig. 1:**
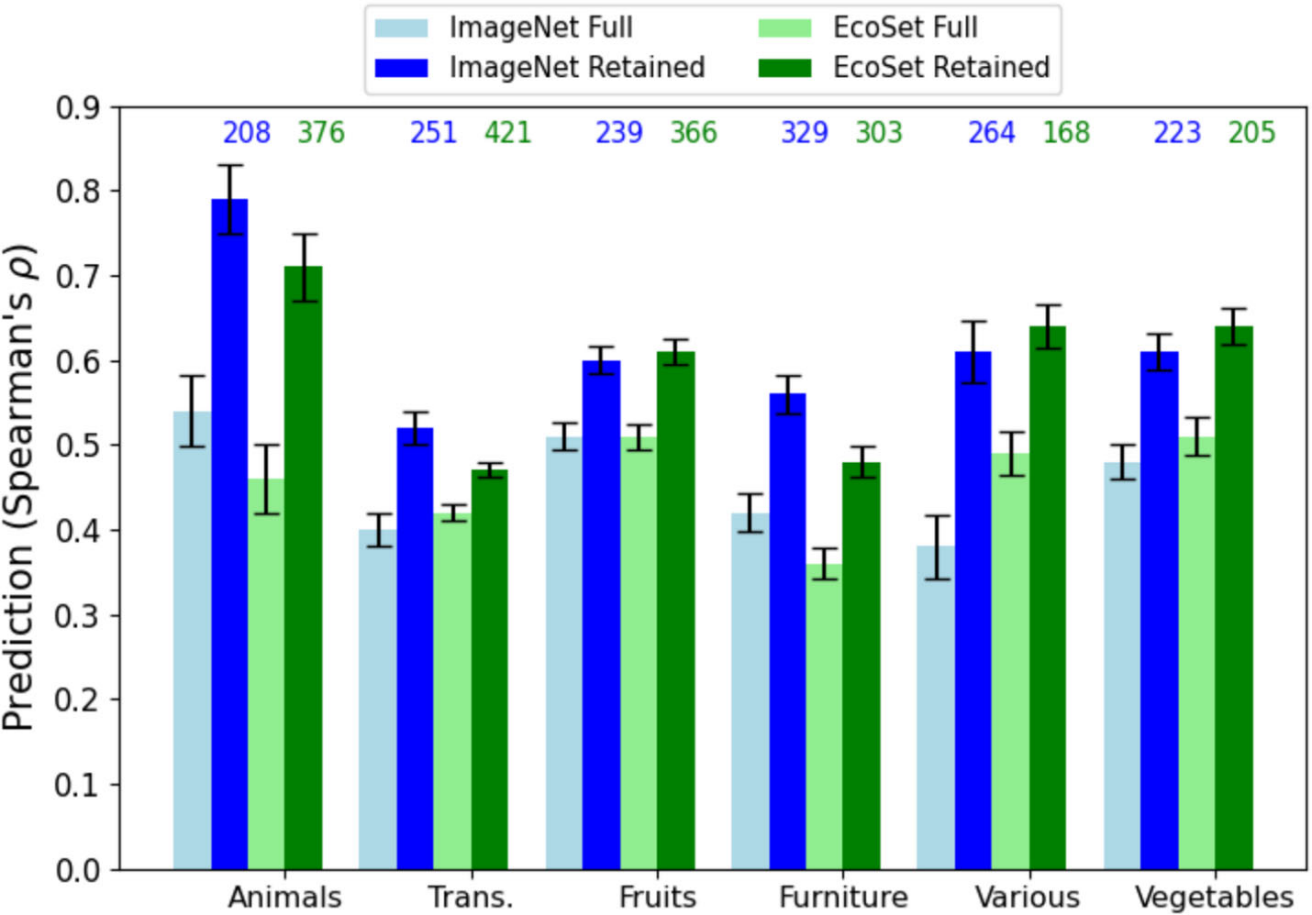
Out-of-sample predictions of human similarity judgments using image embeddings. Full: using all 512 feature maps. Retained: using feature maps identified from the training set. The numbers above the second and fourth columns in each group represent averages of feature map set sizes across 40 folds. Error bars indicate standard errors adjusted for paired-comparisons (Loftus & Masson, 1994)

To evaluate the applicability of the AIS-based analysis to other architectures, we applied the analysis developed for Aim 1, with several modifications, to the following models: inception-V3 (Szegedy et al., 2015), ResNet-152 (He et al., 2016), DenseNet-161 (Huang et al., 2016), EfficientNet-B3 (Tan & Le, 2019), RegNetY-400MF (Radosavovic et al., 2020), and ResNeXt-50-32x4d (Xie et al., 2017). The deepest layers of these architectures contain varying numbers of feature maps: Inception-V3, ResNet-152, and ResNeXt-50-32x4d each have 2,048 feature maps, DenseNet-161 has 2,208, EfficientNet-B3 has 1,536, and RegNetY-400MF has 440. All these architectures are trained on ImageNet classification rather than Ecoset.

We note that all these architectures learn features in the context of supervised classification tasks. To evaluate feature maps produced by non-supervised learning, we used a ResNet-50 architecture trained with the Barlow Twins self-supervised learning framework (Zbontar et al., (2021). In this approach, the objective of the model is to learn representations by maximizing the similarity between two augmented versions of the same image. In this way, training extracts general visual features, ignoring small visual distortions.

For each of these architectures, we performed five-fold cross validation, as detailed for Aim1. For all architectures except VGG-16 and VGG-19, object embeddings were generated by applying global pooling to the feature maps from the deepest convolutional layer. For VGG-16 and VGG-19, embeddings were constructed from the penultimate, fully connected layer.

We also used two additional baselines based on architectures that are already trained to align their representations with human similarity judgments. The first was LPIPS (Learned Perceptual Image Patch Similarity, Zhang et al., 2018), which is a method for obtaining a cognitively-relevant similarity metric between image pairs. LPIPS fine-tunes a pretrained computer vision CNN so that the image distances in the network, calculated as differences between embedding vectors, align with human similarity judgments. LPIPS is fine-tuned using human decision data regarding which of two slightly altered images are closer to an original image, and is based on reweighting all layers of the network. It has been shown to closely match human behavior in 2-alternative forced choice (2AFC) tasks involving minor image distortions and a reference image. The second baseline was DreamSim (Fu et al., 2023), a model that combines information from multiple computer vision models to learn human similarity choices in a 2-AFC task. DreamSim is trained on image triplets where the reference and choice images can differ substantially from each other. This allows it to learn more complex semantics as compared to LPIPS, resulting in improved performance.

To evaluate whether LPIPS is at all viable for our materials and similarity judgments, we applied LPIPS to all images in each dataset to compute pairwise distances between images and computed the Spearman correlation between the LPIPS distance matrix and the human similarity judgments. Note that the LPIPS method does not allow integration with pruning, as its reweighting function achieves a parallel goal. We use the pre-trained LPIPS weights provided by the original authors. We applied the same approach to DreamSim, computing the correlation between the distance matrix produced from DreamSim and human similarity judgments. Furthermore, because the embedding space learned by DreamSim is itself amenable to pruning, we evaluated if its performance is improved when applying our pruning method. To this end, for DreamSim we also performed supervised pruning and evaluation using five-fold cross-validation, as for the other architectures.

**Fig. 2:**
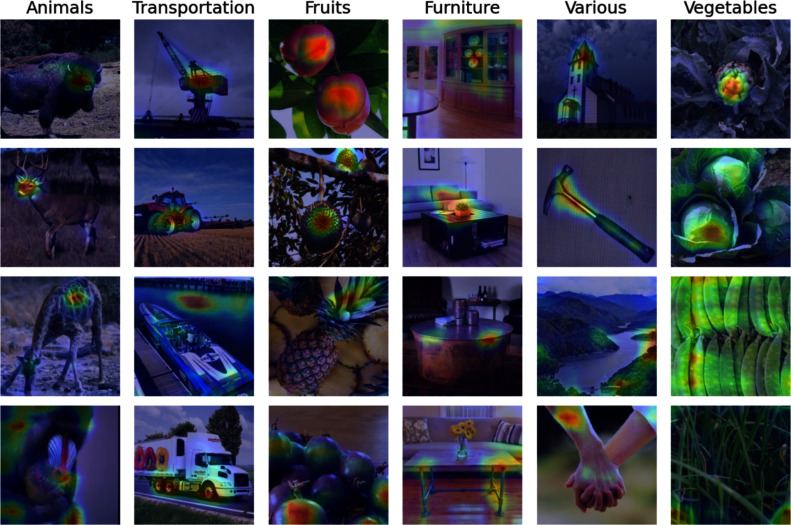
Heatmaps generated using Alignment Importance Scores of feature maps trained with Ecoset. For each dataset, two images with subjectively higher interpretability (top two rows) and lower interpretability (bottom two rows) were selected

## Results

### Aim 1: Identifying a Subset of Feature Maps that Optimizes Prediction of Human Similarity Judgments

As shown in Fig. 1, by computing AIS it was possible to identify a subset of 512 feature maps for each dataset, which produced improved out-of-sample predictions compared to a baseline condition where all feature maps were used. This was consistent for models trained on Ecoset or ImageNet, with less than 50% of the 512 feature maps being used in six of the 12 cases. Paired T-tests indicated that in all 12 cases, predictions from Full features were less accurate than those from features learned via pruning (*p*-values < 0.01). The performance metrics of ImageNet and Ecoset were quite similar. We also repeated this analysis by splitting the train-test sets based on image instances rather than pairwise judgments, so that any single image appeared in either the training set or test set. The results (Appendix Fig. 8) confirm the method’s generalizability, replicating the patterns found here, though more weakly.

Speaking to category-specific information, AIS values for each feature map differed across datasets. That is, feature maps important for aligning one category were not necessarily important for another category. To evaluate this issue, we computed pair-wise Pearson correlations between the AIS values of the 512 feature maps for each pair of datasets (e.g., Fruits vs. Vegetables). Note this analysis uses AIS scores from all feature maps, not only those identified as particularly important by pruning. For both Ecoset and ImageNet, the strongest correlation was between Fruits and Vegetables (Ecoset $r = 0.48$; ImageNet $r = 0.67$). For Ecoset, the second highest correlation was between Transportation and Furniture ($r = 0.38$), whereas for ImageNet it was between Various and Animals ($r = 0.26$). Most of the other correlations, in both analyses, ranged from $-$0.2 to 0.2. This serves as a sanity check, showing that different categories produced different score distributions, while closely related categories, such as Fruits and Vegetables, show high correlations.

**Fig. 3:**
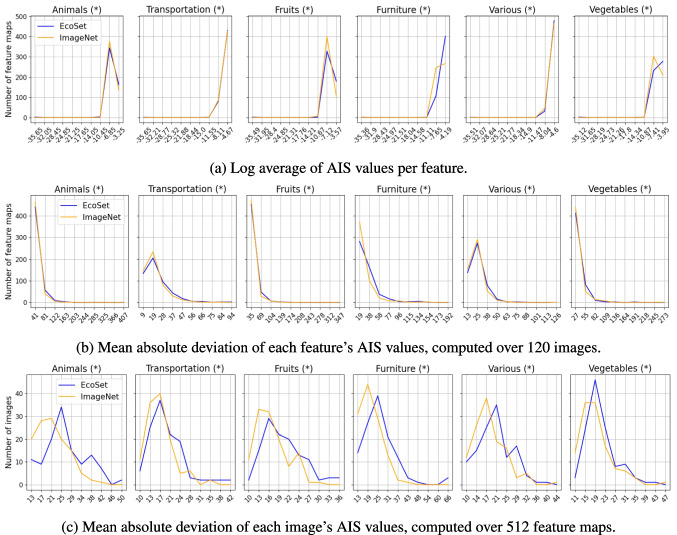
Histograms describing statistics of Alignment Importance Score distributions for models trained on Ecoset or ImageNet. The *x*-axis of **b** and **c** are displayed in e-4 format. An asterisk (*) indicates a significant difference between the two distributions as determined by a KS test

### Aim 2: Explaining Human Similarity Judgments

Figure 2 shows examples of heatmaps produced by alignment importance scoring. Given that each dataset contained 120 images, we selected 4 images from each dataset according to the principle that two of the images produced apparently sensible results, and the two others were less sensible. It can be seen that the method can identify image sections that are relevant for inter-category comparisons, such as the faces of animals, central parts of fruits and vegetables, and discriminating elements of artifacts and man-made objects. As shown subsequently, these are not necessarily the most salient aspects of images.

To assess the similarity of heatmaps produced by Ecoset and ImageNet, for each image we calculated the correlation between the heatmaps produced by the two methods. The median correlation values were as follows: $0.80\pm 0.16$ for Animals, $0.64\pm 0.19$ for Transportation, $0.73\pm 0.22$ for Fruits, $0.64\pm 0.22$ for Furniture, $0.64\pm 0.27$ for Various, and $0.56\pm 0.25$ for Vegetables. In all datasets, the maximum correlation values approached 1.0, while the minimum values often approached zero (see the histogram in Appendix Fig. 9). As Appendix Fig. 9 shows, for all categories (apart from Animals), around 10% of images showed a low correlation of $r <0.2$. Considering $r = 0.8$ as an (arbitrary) reference point for strong correspondence between heatmaps, we find that for Animals more than 40% of the images showed correlations that exceeded this value, whereas for Transportation and Furniture, the value was below 20%.

These results indicate that although agreement was often good, training models on Ecoset or ImageNet often produced different heatmaps. These findings are consistent with those of Aim 1, which showed that the VGG-16 models trained on the two datasets capture and learn human similarity judgments in slightly different ways.

**Fig. 4:**
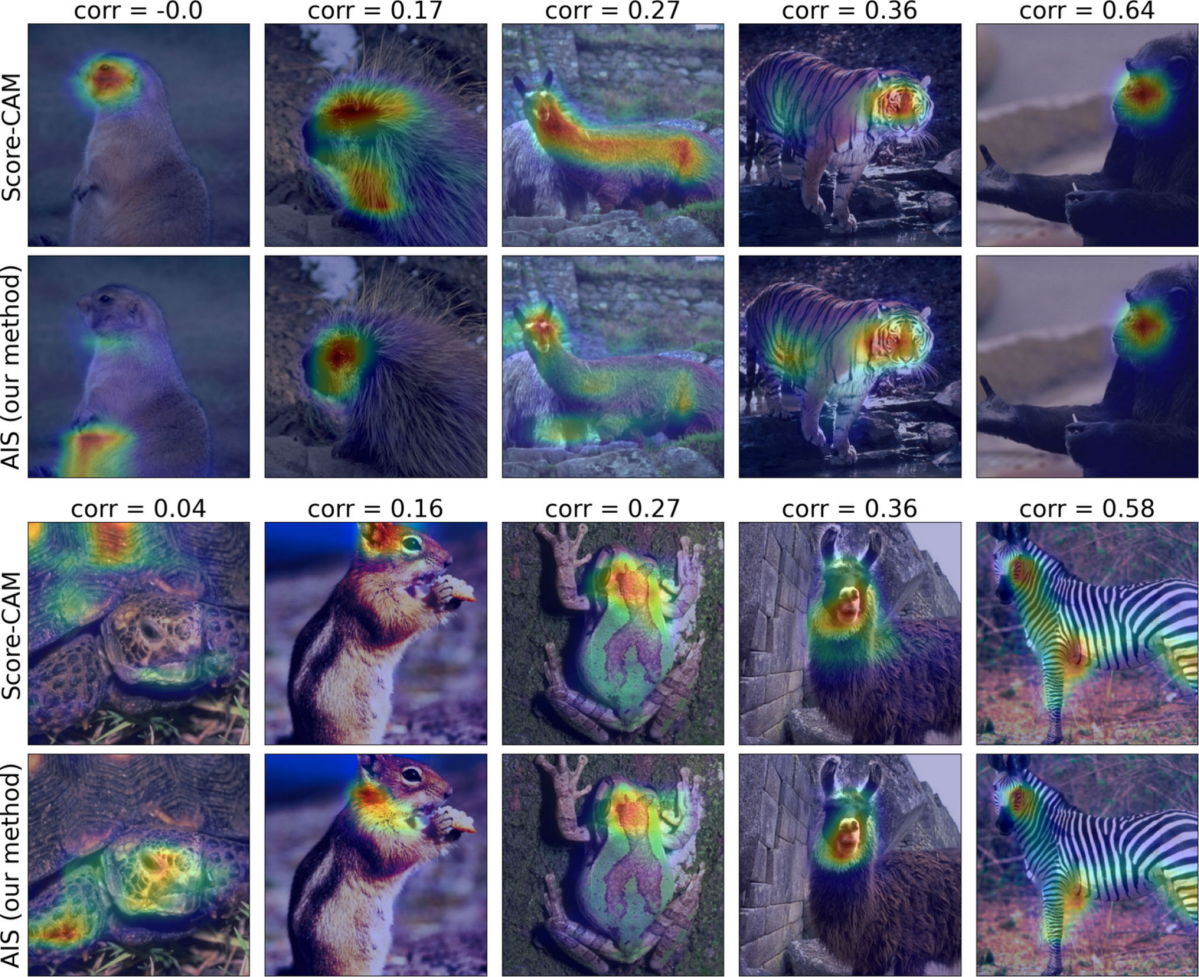
Relation between Score-Cam and AIS-score heatmaps. For each image, we computed the correlation between the importance score assigned by Score-CAM and the one computed by our method. We then selected single images for which these correlations across the two methods ranged from very weak to very strong. The correlation values above each image indicate the correlation values between the two scoring systems for each selected image

As detailed in the “Aim 2: Explaining Human Similarity Judgments” section, we evaluated if images that presented a lower match between Ecoset and ImageNet heatmaps were associated with higher entropy of post-softmax values in either of the two sets (maxEntropy), which would produce a negative correlation between the two quantities. We found that this was indeed the case, for Animals ($r = -0.31$), Fruits ($r = -0.34$), Various ($r = -0.24$), and Vegetables ($r = -0.21$). Weaker, yet still negative correlations were found for Transportation and $-$0.11, Furniture, $rs = -0.11, -0.04$ respectively. Thus, images that do not present information sufficient for classification produce disagreement between the two models. These might be out-of-distribution images or bad examples of trained categories.

Ultimately, in those cases where heatmaps differ, the results of Aim 1 may be used as a guide to inform whether Ecoset or ImageNet is more plausible with respect to the human representation of a given category. For instance, given the low agreement in heatmaps produced for Transportation and Furniture, one may select to use the ImageNet-produced feature maps as these provide better out-of-sample prediction of human behavior.

**Fig. 5:**
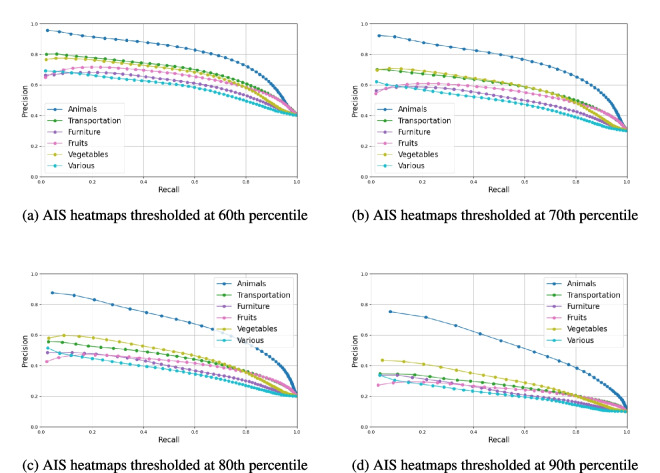
Precision-recall curves when predicting AIS heatmap values from saliency, for different thresholds of AIS heatmaps. The target variable was heatmap values produced from AIS scores computed from ImageNet training. The predicting variable were saliency map values obtained from TranSalNet

We also statistically quantified the relation between AIS values obtained for feature maps when produced from models trained on Ecoset or ImageNet. Figure 3 shows, for each dataset, histograms computing the Average AIS associated with each feature (log10 scaled), and the mean absolute deviation computed per feature (column) and per image (row). The histogram shows that the average AIS rarely exceeded 0.001 for any feature (Fig. 3a). Two-sided Kolmogorov-Smirnov (KS) tests (Hodges Jr, 1958) were conducted to verify if the histograms associated with the two training regimes (ImageNet, Ecoset) came from the same distribution. Overall, the KS test confirmed significant differences for all six categories ($$ p<.05$$).

With respect to mean absolute deviation (MAD), when computed per feature (Fig. 3b), we find that the values varied around one order of magnitude, with a few features showing relatively higher values meaning they were much more important for some images than others. The MAD histograms computed from per-image data indicated that ImageNet’s AIS distribution was consistently left-shifted with respect to Ecoset’s (Fig. 3c). This means that the AIS produced by the Ecoset-trained model is more strongly distributed, suggesting a more meaningful separation between those features relevant for alignment and those that are not. Two-sided Kolmogorov-Smirnov tests on mean absolute deviation verify significant differences between the two models in all cases (all six datasets, KS tests, $$ p<.05$$).

### Aim 3: Cross-Referencing Heatmaps Against Score-CAM and Saliency Maps

#### Relating to Score-CAM

As described in the “Methods” section, for each image, we computed each feature map’s classification-related importance score (CIS) from Score-CAM, and the feature map’s AIS score as computed by our method, and then computed the correlation across the 512 feature maps to determine if CIS and AIS scores correlate. This produced a single correlation score per image

We focused on the 57 Animal images that were correctly classified by VGG-16. For these images, the mean Pearson correlation between CIS and AIS was $r = 0.28$, with the following distribution: minimum $r= 0.0$, 25% quartile $r= 0.17$, 75% quartile $r= 0.37$, and maximum $r= 0.64$. These results indicate that, on average, the correlation was relatively low. Figure 4 contrasts examples of heatmaps generated by Score-CAM and our method at these different correlation levels.

#### Relating to Saliency: Precision-Recall Curves

For each image, we thresholded the AIS-produced heatmap at a given threshold to form a binary prediction target with AIS-related image sections (after thresholding) constituting the positive class. We then evaluated the extent to which these could be predicted by the TranSalNet saliency maps, using a precision-recall curve. In this analysis, the target variable is thresholded at a fixed level (e.g., 90th percentile), while the predicting variable is thresholded across a range of levels, with precision and recall computed for each threshold.

**Table 1:** Relative risk values comparing heatmaps computed from Alignment Importance Scores to those generated by TranSalNet, a saliency model that predicts human gaze

Category	Ecoset			ImageNet		
	5% vs. 5%	10% vs. 10%	15% vs. 15%	5% vs. 5%	10% vs. 10%	15% vs. 15%
Animals	30.8 ± 32.1	17.0 ± 18.3	12.7 ± 11.0	28.2 ± 34.2	14.9 ± 11.5	11.4 ± 8.2
Transportation	7.8 ± 11.5	5.8 ± 7.0	5.2 ± 6.5	6.4 ± 7.8	5.6 ± 5.8	5.3 ± 5.2
Fruits	9.9 ± 18.5	7.4 ± 10.9	6.2 ± 9.2	9.9 ± 21.4	6.6 ± 11.4	5.4 ± 8.2
Furniture	6.1 ± 10.3	5.1 ± 6.2	4.5 ± 4.8	6.5 ± 12.0	5.2 ± 6.5	4.6 ± 4.5
Various	17.3 ± 27.4	10.2 ± 11.0	8.7 ± 8.9	14.4 ± 31.4	8.2 ± 9.9	6.7 ± 7.2
Vegetables	6.4 ± 10.7	4.9 ± 6.8	4.1 ± 4.2	7.1 ± 14.7	5.0 ± 6.8	4.1 ± 4.2
All datasets	13.0 ± 22.1	8.4 ± 11.7	6.9 ± 8.4	12.1 ± 23.8	7.6 ± 9.6	6.2 ± 6.9

Chance values are $RR=1$

**Fig. 6:**
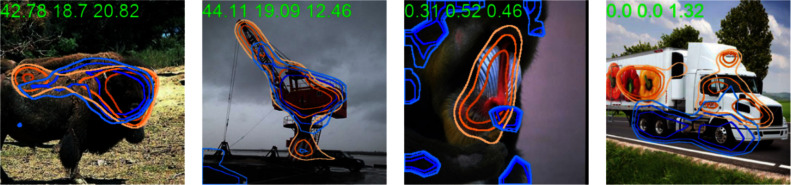
Overlap between the heatmaps created by Alignment Importance Scores (blue contours) and the saliency maps from TranSalNet (orange contours). The contours indicate the 5%, 10%, and 15% most important pixels, with increasing color intensity respectively. Relative risk values computed from top 5%, 10%, and 15% pixels in each map are printed on the top of each image. The two left images are examples of cases where AIS and saliency identified similar areas, whereas the two right images present extreme cases of non-overlap

Figure 5a shows the results when predicting AIS heatmaps produced from ImageNet-trained feature maps, and with the AIS heatmaps thresholded at the 60th percentile. We observe that when saliency maps are thresholded at stringent levels (leftward points on the curve), precision is high for the Animals category, and somewhat lower for other categories, with values ranging from 0.6 to 0.8.

Lowering the threshold increased recall, but also gradually lowered precision as expected, While saliency and AIS maps were clearly related, with the exception of Animals, predicting AIS from saliency appeared limited, even when AIS heatmaps were thresholded at a relatively low value of the 60th percentile. The other panels in Fig. 5 show the same analysis with AIS heatmaps thresholded at the 70th, 80th, and 90th percentiles. In the latter analysis, AIS-relevant pixels are defined as the top 10%, and as shown in Fig. 5d), saliency predicted membership in this class poorly, with the exception of the Animals category. Another observation is that for the Fruits category, thresholding the saliency map at the most strict level (left-most point) did not produce the highest precision, which was instead achieved at lower thresholds. This suggests that the most salient points were not always the most precise predictors of AIS heatmaps.

In summary, we found that, with the exception of the Animals category, saliency heatmaps could not predict AIS heatmaps with good precision and recall, particularly when AIS heatmaps were thresholded at higher levels. A very similar pattern was found for AIS heatmaps produced from Ecoset feature maps (see Appendix Fig. 11).

#### Relating to Saliency: Conditional Probability Analysis

We observed that areas identified as comparison-relevant by AIS heatmaps were much more likely to be associated with salient image sections than with non-salient image sections, as indicated by relative risk values strongly exceeding 1.0 (Table 1). This was found regardless of whether pixels in both heatmaps were thresholded at top 5%, top 10%, or top 15%. As the table shows, the RR values often exceeded 5, reaching as high as 30 for Animals. The data were quite similar for ImageNet and Ecoset overall. Furthermore, the relative risk values varied significantly across categories, being highest for Animals, and lowest for Vegetables. This suggests that for Animals, elements salient in images are also important for comparison, whereas this is less so for Vegetables. This is numerically consistent with the precision-recall analysis where we found that thresholding saliency maps at high percentiles produced good prediction-precision of AIS data.

Figure 6 presents images on which we plotted contours reflecting TranSalNet’s salience (orange) and alignment score heatmaps (blue) to visualize their overlap. For the two images on the left (bison and crane), the salience and alignment maps consistently show strong agreement across all three thresholding levels. For the two right images, there is no overlap. Specifically, the monkey’s facial features are highly salient, but are not identified as important for alignment. In the case of the truck image, the banner area depicting colorful peppers is identified as salient, but the wheel area is identified as important for alignment. This is reasonable, as means of transportation in the set are effectively compared by observing the lower section of the vehicle, which differentiates trucks, cars, buses, motorcycles, trains, and so on. Indeed we find these elements are often highly salient in the produced heatmaps. More results with an appropriate level of detail are shown in the Appendix.

**Fig. 7:**
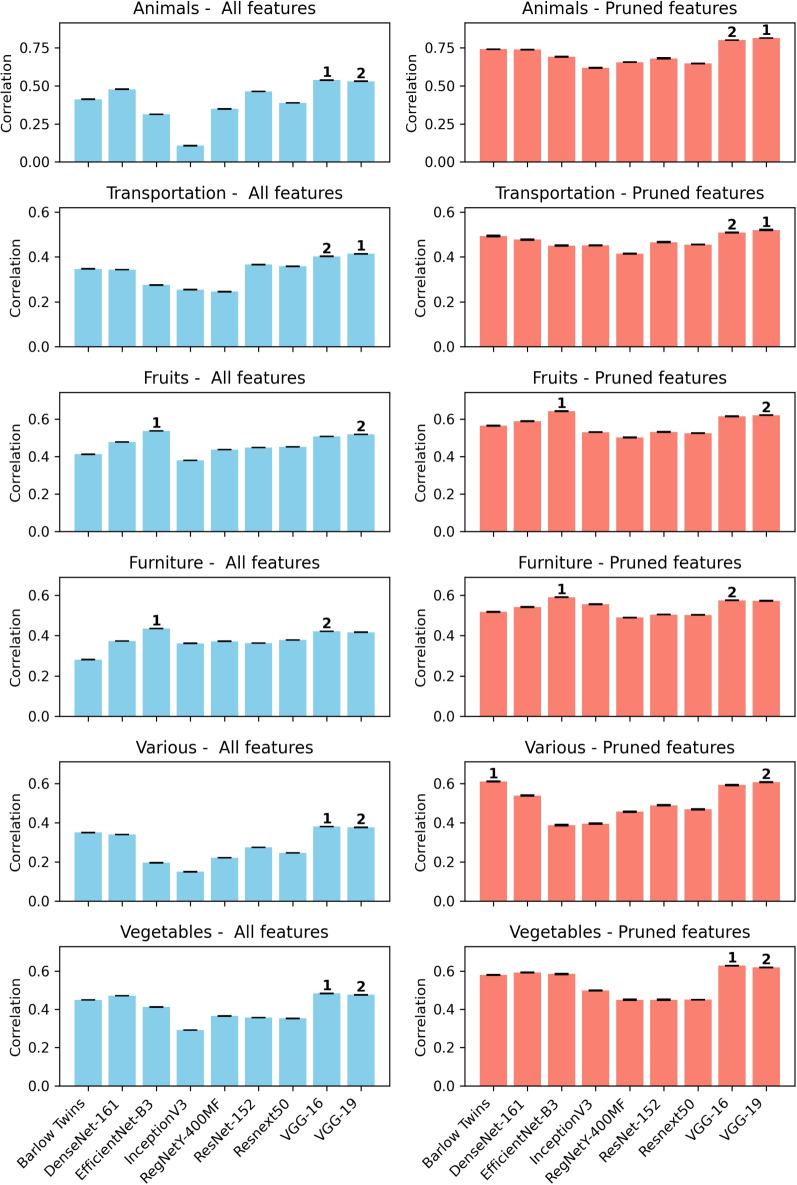
Cross-validation performance for models typically used as feature extractors. The numbers “1” and “2” refer to the two best performing models on the test set when using all features or only the features retained from the training set (Pruned features)

### Aim 4: Generalization to Other Architectures and Training Objectives

We find that quantifying alignment importance and subsequent pruning improved out-of-sample prediction of human similarity judgments across all architectures and all six categories tested (see Fig. 7). Based on the results, we make the following observations. First, the two VGG-based architectures tended to perform best, ranking first in three of the six image categories and second in all six categories. Second, baseline performance (test set prediction using all features) tended to be diagnostic of which architecture would perform best using the learned pruned test set: for four of the six categories, the best performing model when using the full feature sets was also the best performing when using the pruned sets.

However, in three cases, none-VGG models predicted human judgments best. EfficientNet-B3 ranked highest for Fruits and Furniture. The design of this architecture, which aims to balance width, depth, and resolution, has been argued to produce a better representation of relevant image details (see Figure 7 in Tan & Le, 2019). Furthermore, as indicated in the “Methods” section, the fact that this model uses linear combinations of feature map information for classification (after global pooling) makes it potentially more interpretable than VGG-16 and VGG-19, which use fully connected layers to learn complex combinations of feature map information. Finally, the Barlow Twins architecture, which is self-supervised and is not guided by a classification objective, performed the best in the Various category.

For reference, we also evaluated the performance of two methods — LPIPS and DreamSim — that pre-align embeddings from computer vision models to human similarity judgments (see the “Methods” section). We find that LPIPS image distances indeed tracked human similarity judgments for all categories, in that higher LPIPS distances were associated with lower similarity. However, these correlations were quite low. Spearman rho values were Animals 0.15, Automobiles 0.19, Fruits 0.15, Furniture 0.07, Vegetables 0.40, and Various 0.19. Thus, alignment with LPIPS did not approach the levels seen in Fig. 1, even for the non-pruned cases.

In contrast, DreamSim performed very well compared to the nine models not pre-aligned to human data. When compared to the performance of non-pruned models, DreamSim-based embeddings produced the best prediction of human similarity judgments for five of the six categories (with the exception of Animals). This is perhaps not surprising given that this architecture essentially transforms embeddings of multiple pre-trained models to align with human judgments. More interestingly, DreamSim also strongly benefited from additional pruning. For Fruits, Vegetables, Furniture, and Various, pruned DreamSim produced the best prediction (improving over the 2nd-best model by $r=0.01, r= 0.08, r=0.07, r=0.05$ respectively). For Transportation, it matched VGG-19, and only for Animals did it perform less well than VGG-16 and VGG-19.

Given that Peterson et al. (2018) also reported the noise ceiling (computed from inter-participant agreement on similarity judgments), we evaluated how well DreamSim approximated the ceiling. We focused on the four categories for which this model outperformed all others. The resulting values for the four categories were as follows (noise ceiling/pruned model): Fruits 0.75/0.61; Furniture 0.80/0.70; Various 0.83/0.74; Vegetables 0.79/0.71. Thus, DreamSim, when pruned, approximated 80–90% of the noise ceiling across these categories, indicating good alignment with human similarity judgments.

These findings suggest that the VGG architectures show considerable strength overall. However, the impact of removing single feature maps in these architectures is effectively evaluated via the changes in activations in the fully connected layers, which learn interactions between feature maps. Depending on the aims of the analysis, other architectures may be used if such interaction effects are of no interest. Practically, the findings of Aim 4 suggest that when using AIS-based heatmaps as explanations for human comparisons, it is sensible to use an architecture that best predicts these judgments. Importantly, however, our explainability method requires feature map representations for producing heatmaps. Architectures like DreamSim, which do not learn convolutional filters and do not produce feature maps, cannot be used for creating heatmaps. In such cases, pruning can still be applied as shown here, to the single, flat embedding layer learned by the model. This can allow improved prediction of human behavior, but does not allow production of associated heatmaps.

## Discussion

Understanding what information is used in human comparisons is important not only for a better understanding of the comparison process itself, but also for comprehending how people form memories and make decisions (Roads & Love, 2024). We introduced and validated a feature map’s Alignment Importance as a meaningful parameter relevant to such explanations. We first showed that AIS values generalize to improve the prediction of human similarity judgments. This complements current approaches that achieve improvements by using reweighting or pruning of nodes in a DNN’s penultimate layer (e.g., Peterson et al., 2018; Attarian et al., 2020; Kaniuth & Hebart, 2022; Jha et al., 2023; Tarigopula et al., 2023).

We then used AIS to produce explanations for those judgments via heatmaps. These heatmaps offered some correspondence to state-of-the-art saliency maps, in that when saliency maps were thresholded at high percentiles, the resulting representation could sometimes predict (binarized) AIS heatmaps quite well, especially for Animals. However, instances where saliency and AIS-reduced maps diverged are of major theoretical importance as they show it is possible to dissociate visually salient image elements from those that are important for comparison.

We also found that our heatmaps did not always align with those produced by Score-CAM, which is unsurprising given the different objectives of the two methods. Score-CAM quantifies the relative importance of each feature map for the correct classification of an image and will identify features that distinguish one category from others. In contrast, our method, as applied here, identifies dimensions that differentiate objects within a category. The success of pruning applied to the Barlow Twins architecture also shows that pruning can identify relatively low-level perceptual features relevant to human similarity judgments. As opposed to the other architectures examined, whose feature spaces are produced to maximize classification accuracy, the training objective of the Barlow Twins architecture is based on a contrastive loss term that is not informed by category membership, which learns the important visual dimensions of images. The fact that Barlow Twins performed competitively, and was even the best performing model for the Various category, suggests that classification learning is not necessary for producing feature spaces that can be effectively pruned to align with human similarity judgments.

Because the method we present is based on mapping, or aligning a DNN’s representational space to a human one via pruning, the feature space of the pretrained-DNN is of fundamental importance. For this reason, in Aim 1 we studied DNNs trained on both ImageNet and Ecoset datasets. We found that AIS scores improved out-of-sample prediction for models trained on either of the training datasets. Thus, both models learn feature maps particularly relevant for accounting for the representational space of specific categories. For both Ecoset and ImageNet, category-specificity was shown in the fact that the relative ranking of AIS scores varied greatly across categories. Interestingly, Ecoset appears to distribute the AIS scores slightly more uniformly across feature maps than ImageNet, which is a topic that requires further investigation.

Further speaking to generalization across both training sets, the heatmaps were, for the most part, quite similar when created from Ecoset or ImageNet AIS scores, with average correlations between the heatmaps exceeding 0.75 for the Animals category. However, some images showed low correlations, and these tended to be associated with more uniform post-softmax distributions in the DNN’s categorization layer. This means that divergence in heatmaps produced by the two models was more prevalent for images that one of the models found difficult to classify. In practice, we recommend using both Ecoset and ImageNet-trained models to create heatmaps and carefully evaluating images with inconsistent results.

The strongest demonstration of generalization of the AIS-based approach was provided in Aim 4, where we showed that the method improves out-of-sample prediction of human similarity judgments across eight different architectures. From the perspective of construct validity, the choice of architecture is fundamental for the effective use of the proposed method. An architecture that provides poor out-of-sample predictions of human similarity judgments will offer less meaningful explanations of human behavior compared to one that provides strong predictions. Examining this issue we find that there was no architecture that provided the best prediction across all six image categories. Thus, when explaining human comparisons for a stimulus set, it would be generally important to select an architecture with the best predictive capacity.

However, we also note that predictive capacity should be considered conjointly with the complexity of the architecture. In the current study, we used the VGG architecture in Aims 1, 2, and 3, as it was the reference architecture in prior work on prediction of human similarity judgments from image embeddings (Attarian et al., 2020; Peterson et al., 2018; Tarigopula et al., 2023). As mentioned in the “Methods” and “Results” sections, the two VGG architectures, while providing good predictions, produce embeddings that naturally reflect interactions between feature map information, and so the removal of a feature map is assessed by the impact of its removal on these interaction values. Other architectures that do not use fully connected layers after the deepest convolutions may produce simpler explanations. This is a topic that needs to be explored in future work.

## Figures and Tables

**Algorithm 1:**
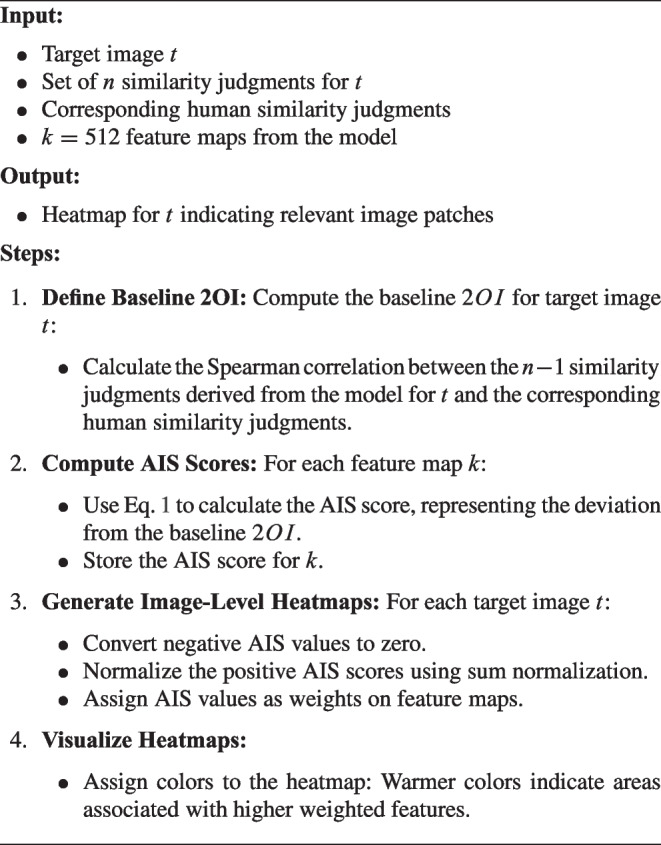
Generate Heatmaps from AIS Scores

**Fig. 8:**
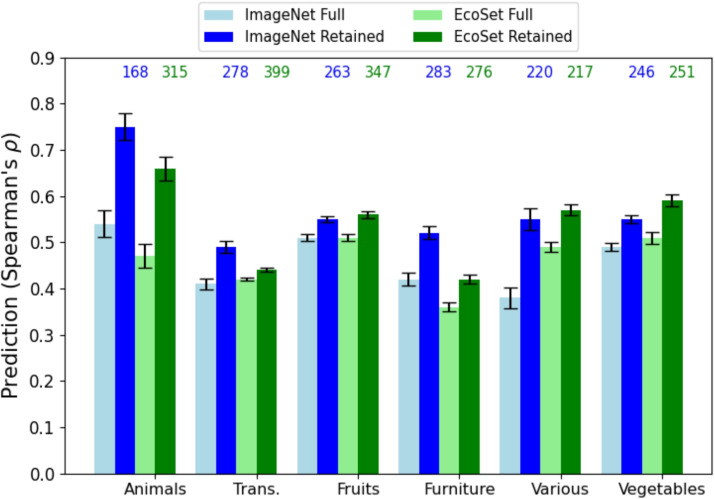
Out-of-sample predictions of human similarity judgments on new images. Here, we used a 3-fold cross-validation framework where 120 images were divided into 80 for training and 40 for testing. Unlike the main Aim 1 analysis, where pairwise judgments were split, this approach ensures that no images appear in both the training and testing sets. This resulted in an approximate 80:20 ratio for the number of similarity pairs, similar to the ratio in Aim 1. Compared to the results in Fig. 1, prediction accuracy was reduced by up to 0.06 Spearman. However, significant improvements were observed across all categories when using the pruned feature maps instead of the full set

**Fig. 9:**
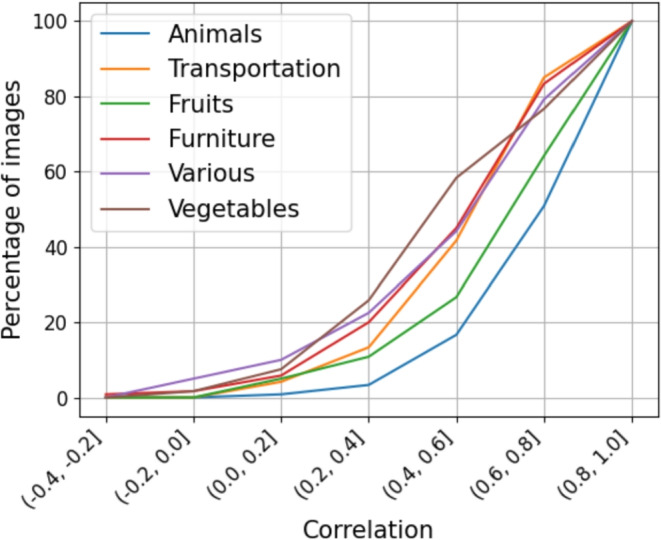
Cumulative histogram of correlations between heatmap’ values created by ImageNet-trained and Ecoset-trained models

**Fig. 10:**
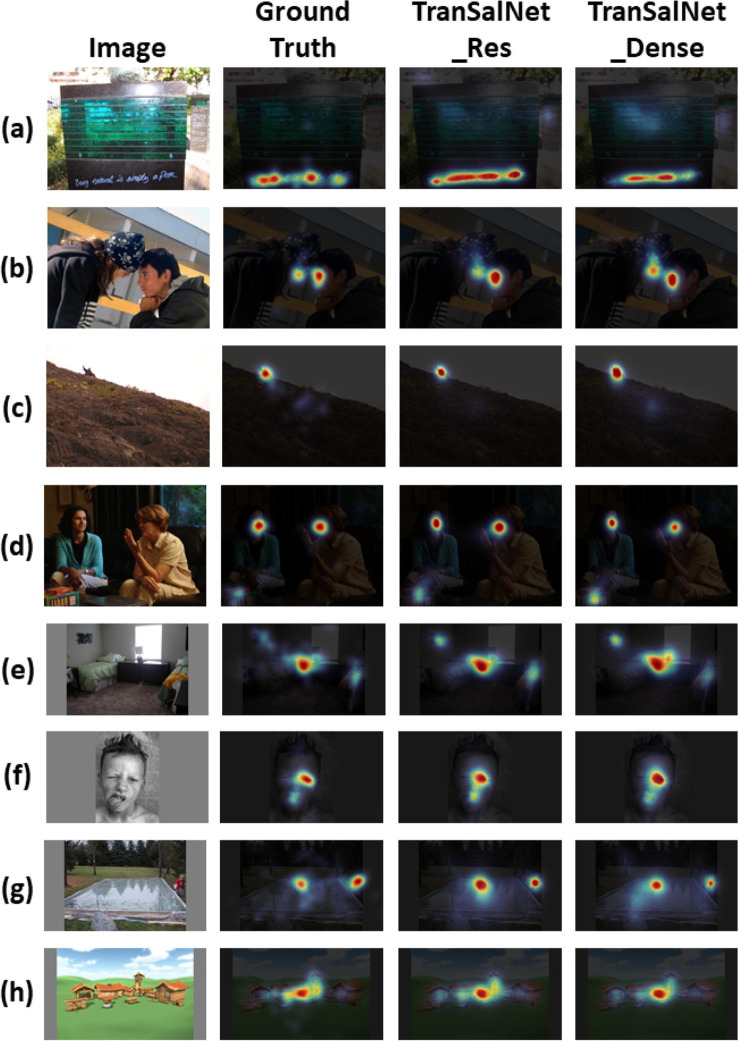
Figure adapted from Lou et al. (2022). https://doi.org/10.1016/j.neucom.2022.04.080. Original figure licensed CC-BY

**Fig. 11:**
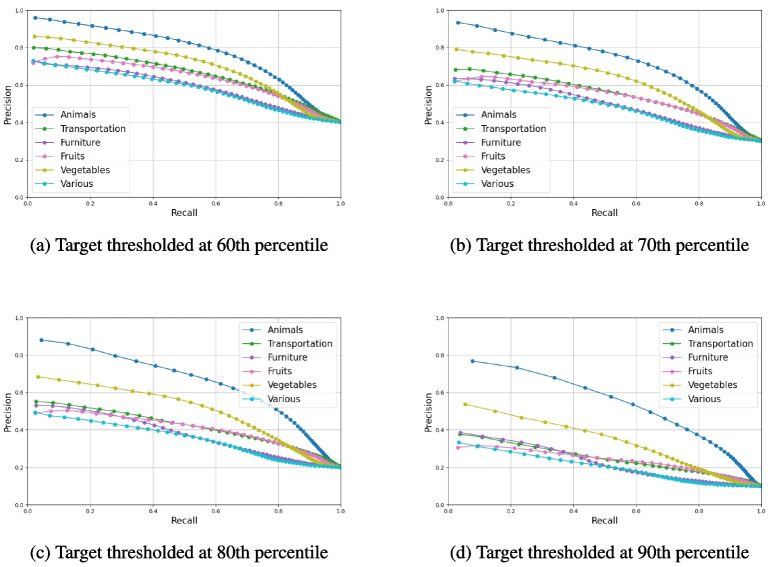
Precision-recall curves for different thresholds of the target variable. The target variable was heatmap values produced from AIS scores computed from Ecoset training. The predicting variable were saliency map values obtained from TranSalNet

## Data Availability

The materials should be requested from the above-mentioned authors. The source code used for generating heatmaps based on image embeddings and corresponding human similarity judgments is publicly accessible at https://github.com/tlmnhut/ais_heatmap.

## Appendix

### A.4 TranSalnet and AIS Maps: Additional Images

Additional images showing the overlap between the heatmaps created by Alignment Importance Scores (blue contours) and the saliency maps from TranSalNet (orange contours). Contours indicate the 5%, 10%, and 15% most important pixels, with increasing color intensity respectively. Relative risk values computed from top 5%, 10%, and 15% pixels in each map are printed on the top of each image.

### A.5 TranSalNet Performance

The image, below, adapted from Lou et al. (2022) shows performance of Translanet in prediction of human gaze. The figure presents the original image, the human gaze location (Ground Truth), and the gaze predictions made by Translanet, when trained on two different vision models.

## Footnotes

1 Available at https://osf.io/kzxfg/

## Code Availability

The code for creating heatmaps can be found at https://github.com/tlmnhut/ais_heatmap
